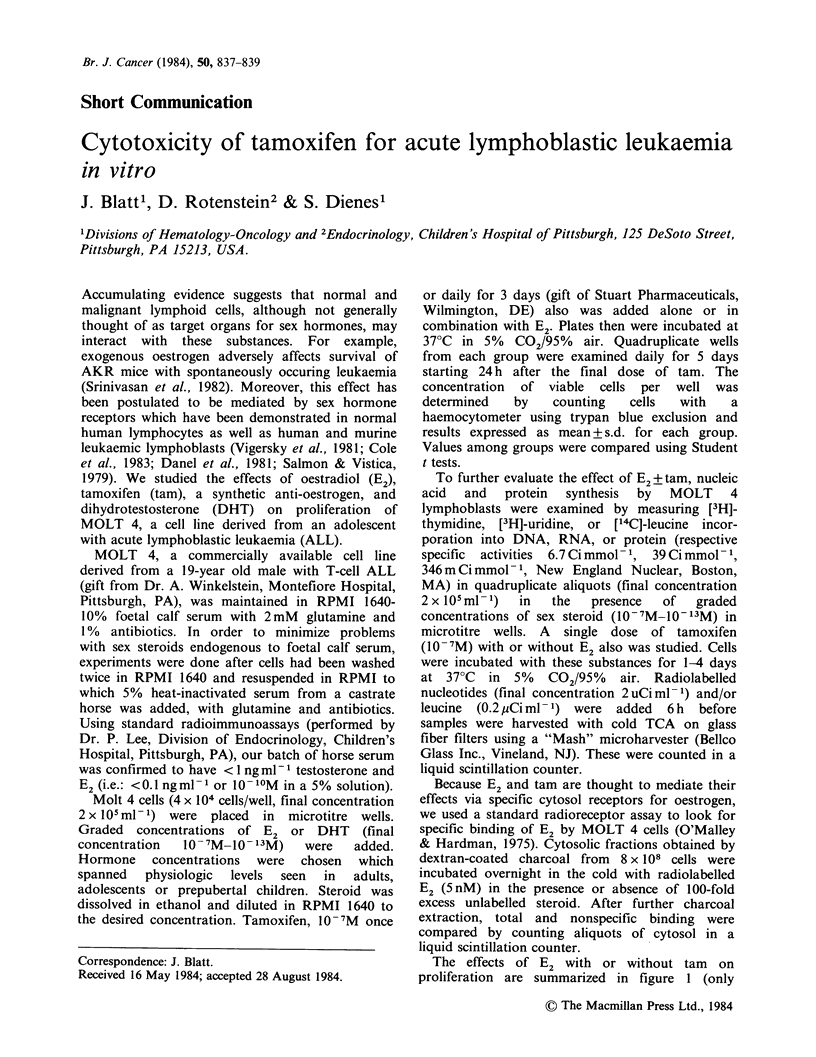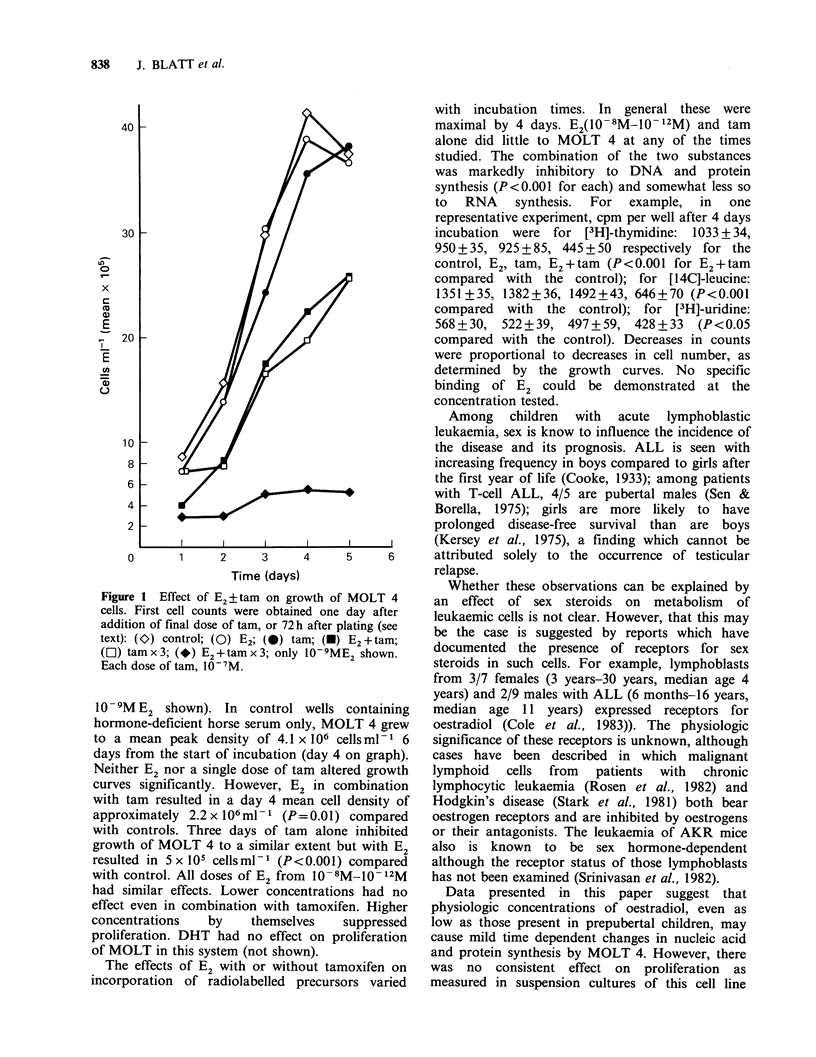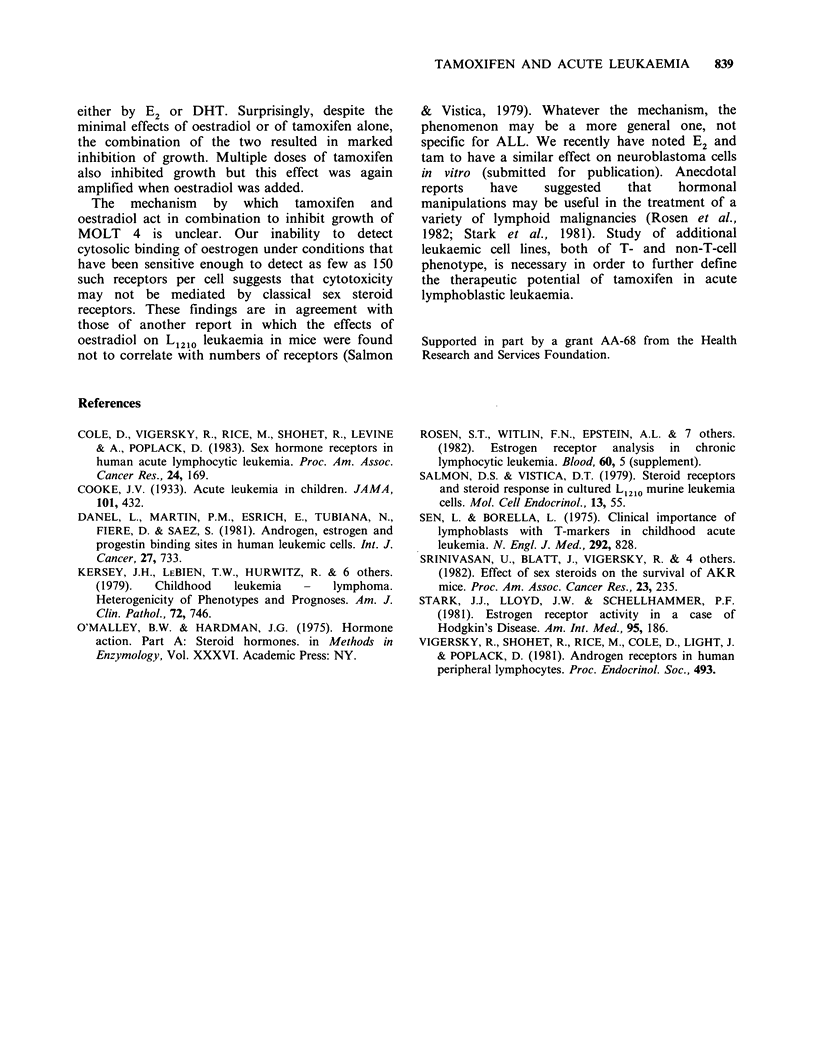# Cytotoxicity of tamoxifen for acute lymphoblastic leukaemia in vitro.

**DOI:** 10.1038/bjc.1984.264

**Published:** 1984-12

**Authors:** J. Blatt, D. Rotenstein, S. Dienes


*Br. J. Cancer* (1984), **50**, 837–839

### Short Communication

### Cytotoxicity of tamoxifen for acute lymphoblastic leukaemia *in vitro*

J. Blatt[1], D. Rotenstein[2] & S. Dienes[1]

[1]*Divisions of Hematology-Oncology and* [2]*Endocrinology, Children's Hospital of Pittsburgh, 125 DeSoto Street, Pittsburgh, PA 15213, USA.*

Accumulating evidence suggests that normal and malignant lymphoid cells, although not generally thought of as target organs for sex hormones, may interact with these substances. For example, exogenous oestrogen adversely affects survival of AKR mice with spontaneously occuring leukaemia (Srinivasan *et al.,* 1982). Moreover, this effect has been postulated to be mediated by sex hormone receptors which have been demonstrated in normal human lymphocytes as well as human and murine leukaemic lymphoblasts (Vigersky *et al.,* 1981; Cole *et al.,* 1983; Danel *et al.,* 1981; Salmon & Vistica, 1979). We studied the effects of oestradiol ($E_2$), tamoxifen (tam), a synthetic anti-oestrogen, and dihydrotestosterone (DHT) on proliferation of MOLT 4, a cell line derived from an adolescent with acute lymphoblastic leukaemia (ALL).

MOLT 4, a commercially available cell line derived from a 19-year old male with T-cell ALL (gift from Dr. A. Winkelstein, Montefiore Hospital, Pittsburgh, PA), was maintained in RPMI 1640-10% foetal calf serum with 2 mM glutamine and 1% antibiotics. In order to minimize problems with sex steroids endogenous to foetal calf serum, experiments were done after cells had been washed twice in RPMI 1640 and resuspended in RPMI to which 5% heat-inactivated serum from a castrate horse was added, with glutamine and antibiotics. Using standard radioimmunoassays (performed by Dr. P. Lee, Division of Endocrinology, Children's Hospital, Pittsburgh, PA), our batch of horse serum was confirmed to have $<1\,\mathrm{ng\,ml^{-1}}$ testosterone and $E_2$ (i.e.: $<0.1\,\mathrm{ng\,ml^{-1}}$ or $10^{-10}$M in a 5% solution).

Molt 4 cells ($4\times10^4$ cells/well, final concentration $2\times10^5\,\mathrm{ml^{-1}}$) were placed in microtitre wells. Graded concentrations of $E_2$ or DHT (final concentration $10^{-7}$M–$10^{-13}$M) were added. Hormone concentrations were chosen which spanned physiologic levels seen in adults, adolescents or prepubertal children. Steroid was dissolved in ethanol and diluted in RPMI 1640 to the desired concentration. Tamoxifen, $10^{-7}$M once or daily for 3 days (gift of Stuart Pharmaceuticals, Wilmington, DE) also was added alone or in combination with $E_2$. Plates then were incubated at 37°C in 5% $CO_2$/95% air. Quadruplicate wells from each group were examined daily for 5 days starting 24 h after the final dose of tam. The concentration of viable cells per well was determined by counting cells with a haemocytometer using trypan blue exclusion and results expressed as mean$\pm$s.d. for each group. Values among groups were compared using Student *t* tests.

To further evaluate the effect of $E_2\pm$tam, nucleic acid and protein synthesis by MOLT 4 lymphoblasts were examined by measuring [$^3$H]-thymidine, [$^3$H]-uridine, or [$^{14}$C]-leucine incorporation into DNA, RNA, or protein (respective specific activities $6.7\,\mathrm{Ci\,mmol^{-1}}$, $39\,\mathrm{Ci\,mmol^{-1}}$, $346\,\mathrm{mCi\,mmol^{-1}}$, New England Nuclear, Boston, MA) in quadruplicate aliquots (final concentration $2\times10^5\,\mathrm{ml^{-1}}$) in the presence of graded concentrations of sex steroid ($10^{-7}$M–$10^{-13}$M) in microtitre wells. A single dose of tamoxifen ($10^{-7}$M) with or without $E_2$ also was studied. Cells were incubated with these substances for 1–4 days at 37°C in 5% $CO_2$/95% air. Radiolabelled nucleotides (final concentration $2\,\mathrm{uCi\,ml^{-1}}$) and/or leucine ($0.2\,\mu\mathrm{Ci\,ml^{-1}}$) were added 6 h before samples were harvested with cold TCA on glass fiber filters using a "Mash" microharvester (Bellco Glass Inc., Vineland, NJ). These were counted in a liquid scintillation counter.

Because $E_2$ and tam are thought to mediate their effects via specific cytosol receptors for oestrogen, we used a standard radioreceptor assay to look for specific binding of $E_2$ by MOLT 4 cells (O'Malley & Hardman, 1975). Cytosolic fractions obtained by dextran-coated charcoal from $8\times10^8$ cells were incubated overnight in the cold with radiolabelled $E_2$ (5 nM) in the presence or absence of 100-fold excess unlabelled steroid. After further charcoal extraction, total and nonspecific binding were compared by counting aliquots of cytosol in a liquid scintillation counter.

The effects of $E_2$ with or without tam on proliferation are summarized in figure 1 (only

Correspondence: J. Blatt.
Received 16 May 1984; accepted 28 August 1984.

© The Macmillan Press Ltd., 1984

**Figure 1** Effect of $E_2 \pm$tam on growth of MOLT 4 cells. First cell counts were obtained one day after addition of final dose of tam, or 72 h after plating (see text): (◇) control; (○) $E_2$; (●) tam; (■) $E_2$+tam; (□) tam × 3; (◆) $E_2$+tam × 3; only $10^{-9}$M$E_2$ shown. Each dose of tam, $10^{-7}$M.

$10^{-9}$M $E_2$ shown). In control wells containing hormone-deficient horse serum only, MOLT 4 grew to a mean peak density of $4.1 \times 10^6$ cells ml$^{-1}$ 6 days from the start of incubation (day 4 on graph). Neither $E_2$ nor a single dose of tam altered growth curves significantly. However, $E_2$ in combination with tam resulted in a day 4 mean cell density of approximately $2.2 \times 10^6$ ml$^{-1}$ ($P = 0.01$) compared with controls. Three days of tam alone inhibited growth of MOLT 4 to a similar extent but with $E_2$ resulted in $5 \times 10^5$ cells ml$^{-1}$ ($P < 0.001$) compared with control. All doses of $E_2$ from $10^{-8}$M–$10^{-12}$M had similar effects. Lower concentrations had no effect even in combination with tamoxifen. Higher concentrations by themselves suppressed proliferation. DHT had no effect on proliferation of MOLT in this system (not shown).

The effects of $E_2$ with or without tamoxifen on incorporation of radiolabelled precursors varied with incubation times. In general these were maximal by 4 days. $E_2$($10^{-8}$M–$10^{-12}$M) and tam alone did little to MOLT 4 at any of the times studied. The combination of the two substances was markedly inhibitory to DNA and protein synthesis ($P < 0.001$ for each) and somewhat less so to RNA synthesis. For example, in one representative experiment, cpm per well after 4 days incubation were for [$^3$H]-thymidine: $1033 \pm 34$, $950 \pm 35$, $925 \pm 85$, $445 \pm 50$ respectively for the control, $E_2$, tam, $E_2$+tam ($P < 0.001$ for $E_2$+tam compared with the control); for [14C]-leucine: $1351 \pm 35$, $1382 \pm 36$, $1492 \pm 43$, $646 \pm 70$ ($P < 0.001$ compared with the control); for [$^3$H]-uridine: $568 \pm 30$, $522 \pm 39$, $497 \pm 59$, $428 \pm 33$ ($P < 0.05$ compared with the control). Decreases in counts were proportional to decreases in cell number, as determined by the growth curves. No specific binding of $E_2$ could be demonstrated at the concentration tested.

Among children with acute lymphoblastic leukaemia, sex is know to influence the incidence of the disease and its prognosis. ALL is seen with increasing frequency in boys compared to girls after the first year of life (Cooke, 1933); among patients with T-cell ALL, 4/5 are pubertal males (Sen & Borella, 1975); girls are more likely to have prolonged disease-free survival than are boys (Kersey *et al.*, 1975), a finding which cannot be attributed solely to the occurrence of testicular relapse.

Whether these observations can be explained by an effect of sex steroids on metabolism of leukaemic cells is not clear. However, that this may be the case is suggested by reports which have documented the presence of receptors for sex steroids in such cells. For example, lymphoblasts from 3/7 females (3 years–30 years, median age 4 years) and 2/9 males with ALL (6 months–16 years, median age 11 years) expressed receptors for oestradiol (Cole *et al.*, 1983)). The physiologic significance of these receptors is unknown, although cases have been described in which malignant lymphoid cells from patients with chronic lymphocytic leukaemia (Rosen *et al.*, 1982) and Hodgkin's disease (Stark *et al.*, 1981) both bear oestrogen receptors and are inhibited by oestrogens or their antagonists. The leukaemia of AKR mice also is known to be sex hormone-dependent although the receptor status of those lymphoblasts has not been examined (Srinivasan *et al.*, 1982).

Data presented in this paper suggest that physiologic concentrations of oestradiol, even as low as those present in prepubertal children, may cause mild time dependent changes in nucleic acid and protein synthesis by MOLT 4. However, there was no consistent effect on proliferation as measured in suspension cultures of this cell line either by $E_2$ or DHT. Surprisingly, despite the minimal effects of oestradiol or of tamoxifen alone, the combination of the two resulted in marked inhibition of growth. Multiple doses of tamoxifen also inhibited growth but this effect was again amplified when oestradiol was added.

The mechanism by which tamoxifen and oestradiol act in combination to inhibit growth of MOLT 4 is unclear. Our inability to detect cytosolic binding of oestrogen under conditions that have been sensitive enough to detect as few as 150 such receptors per cell suggests that cytotoxicity may not be mediated by classical sex steroid receptors. These findings are in agreement with those of another report in which the effects of oestradiol on $L_{1210}$ leukaemia in mice were found not to correlate with numbers of receptors (Salmon & Vistica, 1979). Whatever the mechanism, the phenomenon may be a more general one, not specific for ALL. We recently have noted $E_2$ and tam to have a similar effect on neuroblastoma cells *in vitro* (submitted for publication). Anecdotal reports have suggested that hormonal manipulations may be useful in the treatment of a variety of lymphoid malignancies (Rosen *et al.*, 1982; Stark *et al.*, 1981). Study of additional leukaemic cell lines, both of T- and non-T-cell phenotype, is necessary in order to further define the therapeutic potential of tamoxifen in acute lymphoblastic leukaemia.

Supported in part by a grant AA-68 from the Health Research and Services Foundation.

### References

COLE, D., VIGERSKY, R., RICE, M., SHOHET, R., LEVINE & A., POPLACK, D. (1983). Sex hormone receptors in human acute lymphocytic leukemia. *Proc. Am. Assoc. Cancer Res.*, **24**, 169.

COOKE, J.V. (1933). Acute leukemia in children. *JAMA*, **101**, 432.

DANEL, L., MARTIN, P.M., ESRICH, E., TUBIANA, N., FIERE, D. & SAEZ, S. (1981). Androgen, estrogen and progestin binding sites in human leukemic cells. *Int. J. Cancer*, **27**, 733.

KERSEY, J.H., LEBIEN, T.W., HURWITZ, R. & 6 others. (1979). Childhood leukemia – lymphoma. Heterogenicity of Phenotypes and Prognoses. *Am. J. Clin. Pathol.*, **72**, 746.

O'MALLEY, B.W. & HARDMAN, J.G. (1975). Hormone action. Part A: Steroid hormones. in *Methods in Enzymology*, Vol. XXXVI. Academic Press: NY.

ROSEN, S.T., WITLIN, F.N., EPSTEIN, A.L. & 7 others. (1982). Estrogen receptor analysis in chronic lymphocytic leukemia. *Blood*, **60**, 5 (supplement).

SALMON, D.S. & VISTICA, D.T. (1979). Steroid receptors and steroid response in cultured $L_{1210}$ murine leukemia cells. *Mol. Cell Endocrinol.*, **13**, 55.

SEN, L. & BORELLA, L. (1975). Clinical importance of lymphoblasts with T-markers in childhood acute leukemia. *N. Engl. J. Med.*, **292**, 828.

SRINIVASAN, U., BLATT, J., VIGERSKY, R. & 4 others. (1982). Effect of sex steroids on the survival of AKR mice. *Proc. Am. Assoc. Cancer Res.*, **23**, 235.

STARK, J.J., LLOYD, J.W. & SCHELLHAMMER, P.F. (1981). Estrogen receptor activity in a case of Hodgkin's Disease. *Am. Int. Med.*, **95**, 186.

VIGERSKY, R., SHOHET, R., RICE, M., COLE, D., LIGHT, J. & POPLACK, D. (1981). Androgen receptors in human peripheral lymphocytes. *Proc. Endocrinol. Soc.*, **493.**